# Supplementary material for: Deciphering the West Eurasian Genetic Footprints in Ancient South India
Source: Genes (Basel). 2023 Apr 23;14(5):963. doi: 10.3390/genes14050963 (PMC10218305; doi:10.3390/genes14050963)
Supplement: Supplementary file 1 [file genes-14-00963-s001.zip › genes-2293510-supplementary.pdf]

Supplementary material

Deciphering the West Eurasian Genetic Footprints in Ancient South India

Bhavna Ahlawat [1§] · Lomous Kumar[2§] · P J Cherian[3, 4] · J S Sehrawat[1] · Niraj Rai[5*] · Kumarasamy Thangaraj [2, 6*]

[1] Department of Anthropology, Panjab University, Chandigarh, 160014, India

[2] CSIR-Centre for Cellular and Molecular Biology, Uppal Road, Hyderabad 500007, India

[3] PAMA Institute for the Advancement of Transdisciplinary Archaeological Sciences, Pattanam Archaeological Site, 683522, India.

[4] Kerala Council for Historical Research & Director Pattanam Excavations

[5] Birbal Sahni Institute of Palaeosciences, Lucknow, 226007, India

[6] Centre for DNA Fingerprinting and Diagnostics, Uppal, Hyderabad 500007, India

Table S1: Genotype data from 95 ancestry mtDNA markers:

mtDNA mutation sites		Haplogroups: HV	T1a9	JT	HV4b	M2a1a3	M6	R1	M3a1	R1	U1	UC	H3Z1
		Sample codes											
	CRS	PT1	PT2	PT3	PT4	PT5	PT6	PT7	PT8	PT9	PT10	PT11	PT13
2706	G	-	-	-	-	-	-	-	-	-	-	-	-
13167	A	A	A	A		A	A	A	A	-	-	A	A
9540	C	CT	T	CT	C	C	C	CT	C	C	C	-	C
3543	C	C	C	C	C	C	C	C	C	-	C	C	C
3010	G	G	G	G	A	G	G	G	G	-	-	-	G
1524	A	A	A	A	A	A	A	A	A	A	A	A	A
14800	C	C	C	-	-	-	-	-	-	-	-	-	-
7094	T	T	T	T	T	T	T	T	-	-	-	T	-
13710	A	-	A	A	-	A	-	-	-	-	-	-	-
12133	C	C	C	C	-	C	C	C	-	C	-	C	-
2045	A	A	A	A	A	A	A	A	-	A	-	A	A
3384	A	A	A	A	-	A	-	-	A	-	-	A	-
15607	A	A	A	A		A	-	A	A	GA	A	A	-
7859	G	G	G	G	G	G	G	G	G	-	G	G	-
12879	T	T	C	C	T	T	T	T	-	-	C	T	T
16344	C	C	C	C		C			C	-	-	C	-
482	T	T	T	T	T	C	C	T	-	-	-	T	-
16274	G	G	G	G	G	G	G	G	-	-	-	-	-
16111	C	C	T	T	-	C	-	-	-	-	C	C	-
12612	A	A	A	A		A	A	A	A	-	-	A	A
15754	C	C	C	C	C	C	C	C	-	-	-	C	C
8697	G	G	G	G	-	G	G	G	G	-	-	G	G
5783	G	G	G	G	-	G	G	G	-	-	G	G	-
10727	C	C	C	C	C	C	C	C	C	-	-	C	-
11215	C	C	C	C	-	C	-	-	-	-	-	C	-
1453	A	A	A	A	-	A	-		-	-	-	A	-
4065	A	-	-	-	-	-	-	-	-	-	-	-	-
207	G	G	G	G	G	A	-	-	-	-	G	G	-
13263	A	A	A	A	-	A	A	A	A	-	-	A	A
12705	T	C	C	C	-	T	-	-	-	-	-	-	C
13359	G	G	G	G	-	G	G	G	G	-	-	G	-
8502	A	A	A	A	-	A	-	-	-	-	-	-	-
5178	C	C	C	-	-	C	-	C	C	-	-	-	CA
8414	C	C	C	C	C	C	C	C	-	-	-	C	-
4703	T	T	-	T	T	C	-	T	C	-	-	-	-
14905	G	G	G	G	-	G	G	G	-	-	-	G	G
13135	G	G	G	G	-	G	G	G	G	-	G	G	AG
6305	G	G	G	G	-	G	G	G	G	-	-	G	G
11176	G	G	G	G	-	G	G	G	G	-	G	G	-

12950	A	A	A	-	-	A	A	A	A	-	A	A	A
4140	C	C	C	C	-	C	C	C	C	C	-	C	-
12406	G	G	G	G	G	G	-	G	G	G	-	G	-
12618	G	G	G	G	G	G	G	G	G	-	-	G	-
3434	A	A	A	-	A	A	GA	A	A	A	-	A	-
14766	T	C	T	CT	-	T	T	CT	T	T	T	T	T
16069	C	C	C	C	T	C	C	C	C	C	-	C	C
8701	G	A	G	A	A	G	G	A	G	-	A	-	GA
8887	A	GA	GA	G	GA	GA	GA	G	GA	G	G	GA	GA
11719	A	G	A	A	G	A	A	AG	A	-	-	A	-
16288	T	T	T	T	T	T	T	T	T	T	T	-	T
13928	G	G	G	G	-	G	-	G	G	-	G	CG	G
14569	G	G	G	G	G	G	G	G	G	-	-	G	G
15452	C	C	-	C	-	C	CA	C	C	-	-	C	C
16172	T	T	T	T	T	T	T	T	T	T	-	-	T
4775	A	A	A	A	-	A	A	-	A	A	-	A	A
9064	G	G	G	-	-	G	-	-	G	-	-	G	G
12285	T	T	T	T	-	T	T	T	T	-	-	T	-
13759	G	G	G	AG	AG	G	AG	AG	G	AG	AG	G	G
10609	T	T	T	CT	-	T	T	T	T	CT	C	T	-
5301	A	A	A	-	-	A	G	A	A	-	G	A	-
3348	A	A	A	-	A	A	A	A	A	-	-	A	A
9180	A	A	A	A	A	A	A	A	A	A	A	A	A
4907	T	T	T	T	T	T	T	T	T	-	-	-	T
1736	A	A	A	A	A	A	-	A	A	A	-	A	-
249	DEL	A	A	A	-	A	-	A	A	-	-	A	A
1442	G	G	G	G		G	-	G	G	G	-	G	-
1541	T	T	T	T	T	T	-	T	T	T	-	T	-
9581	T	T	T	T	-	T	-	T	-	-	T	T	-
12189	T	T	T	-	-	T	T	T	-	T	T	T	-
12361	A	A	A	-	-	A	A	-	-	-	A	A	-
16223	T	C	T	-	-	T	T	CT	T	C	T	CT	C
2755	A	A	A	-	-	A	-	A	-	-	A	A	-
13104	A	A	A	A	A	A	A	A	-	-	A	A	A
149		T	T	T	-	T	T	-	-	-	T	T	-
16108	C	C	C	-	-	C	-	C	C	-	C	C	C
8014	A	A	A	-	A	A	A	A	-	-	A	A	A
12633	C	C	C	-	C	C	C	C	-	-	C	C	-
4086	C	C	C	-	-	C	C	C	C	-	C	C	C
15440	T	T	T	-	T	T	T	T	T	-	T	T	-
3360	A	A	A	-	A	A	-	-	A	-	A	A	A
11023	A	A	A	-	A	A	A	-	-	A	A	A	
12810	A	A	A	-	A	A	-	-	-	-	A	A	-
3714	A	A	A	-	-	A	-	-	-	-	-	-	-
15530	T	TC	T	C	C	T	TC	C	-	TC	TC	TC	C

13188	C	C	C	TC	-	C	C	C	-	-	C	C	-
1811	A	A	A	-	A	A	-	A	-	A	A	A	A
6620	T	T	T	-	-	T	T	T	-	-	T	T	-
5823	A	A	A	A	A	A	-	A	-	A	A	A	-
1598	G	G	G	-	-	G	-	G	-	G	G	G	-
16294	C	C	C	-	C	C	C	-	C	-	C	C	-
3817	C	C	C	C	C	C	C	C	C	-	C	C	-
11251	A	GA	G	-	-	A	-	-	-	-	A	-	-
16327	C	C	C	C	C	C	C	C	C	C	C	C	C

- Highlighted yellow color bases are the observe SNP in the samples.

Table S2: Conventional and Calibrated Radiocarbon age estimates for three Pattanam samples

Sample Data	Method	Material/ Pretreatment	Measured Radiocarbon Age	13C/12C Ratio	Conventional Radiocarbon Age(*)
PT-03	AMS-Standard delivery	(cremated bone carbonate): bone carbonate extraction	2080 +/- 30 BP	-24.0 o/oo	2100 +/- 30 BP
PT-04	AMS-Standard delivery	(cremated bone carbonate): bone carbonate extraction	1990 +/- 30 BP	-8.7 o/oo	2260 +/- 30 BP
PT- 05	AMS-Standard delivery	(cremated bone carbonate): bone carbonate extraction	2130 +/- 30 BP	-9.2 o/oo	2390 +/- 30 BP

*Database used: INTCAL13

References

Mathematics used for calibration scenario
A Simplified Approach to Calibrating C14 Dates, Talma, A. S., Vogel, J. C., 1993, Radiocarbon 35(2):317-322
References to INTCAL13 database
Reimer PJ et al. IntCal13 and Marine13 radiocarbon age calibration curves 0–50,000 years cal BP. Radiocarbon 55(4):1869–1887.
